# Protective Effects of Myricetin on Acute Hypoxia-Induced Exercise Intolerance and Mitochondrial Impairments in Rats

**DOI:** 10.1371/journal.pone.0124727

**Published:** 2015-04-28

**Authors:** Dan Zou, Peng Liu, Ka Chen, Qi Xie, Xinyu Liang, Qian Bai, Qicheng Zhou, Kai Liu, Ting Zhang, Jundong Zhu, Mantian Mi

**Affiliations:** Research Center for Nutrition and Food Safety, Institute of Military Preventive Medicine, Third Military Medical University; Chongqing Key Laboratory of Nutrition and Food Safety, Chongqing Medical Nutrition Research Center, Chongqing, PR China; West Virginia University School of Medicine, UNITED STATES

## Abstract

**Purpose:**

Exercise tolerance is impaired in hypoxia. The aim of this study was to evaluate the effects of myricetin, a dietary flavonoid compound widely found in fruits and vegetables, on acute hypoxia-induced exercise intolerance in vivo and in vitro.

**Methods:**

Male rats were administered myricetin or vehicle for 7 days and subsequently spent 24 hours at a barometric pressure equivalent to 5000 m. Exercise capacity was then assessed through the run-to-fatigue procedure, and mitochondrial morphology in skeletal muscle cells was observed by transmission electron microscopy (TEM). The enzymatic activities of electron transfer complexes were analyzed using an enzyme-linked immuno-sorbent assay (ELISA). mtDNA was quantified by real-time-PCR. Mitochondrial membrane potential was measured by JC-1 staining. Protein expression was detected through western blotting, immunohistochemistry, and immunofluorescence.

**Results:**

Myricetin supplementation significantly prevented the decline of run-to-fatigue time of rats in hypoxia, and attenuated acute hypoxia-induced mitochondrial impairment in skeletal muscle cells *in vivo* and *in vitro* by maintaining mitochondrial structure, mtDNA content, mitochondrial membrane potential, and activities of the respiratory chain complexes. Further studies showed that myricetin maintained mitochondrial biogenesis in skeletal muscle cells under hypoxic conditions by up-regulating the expressions of mitochondrial biogenesis-related regluators, in addition, AMP-activated protein kinase(AMPK) plays a crucial role in this process.

**Conclusions:**

Myricetin may have important applications for improving physical performance under hypoxic environment, which may be attributed to the protective effect against mitochondrial impairment by maintaining mitochondrial biogenesis.

## Introduction

Mitochondria are the major site of cellular energy production in the form of ATP in mammalian cells[1], and skeletal muscle mitochondrial content is an important determinant of endurance capacity[2]. Previous studies have demonstrated that acute and severe hypobaric hypoxia could increase oxidative stress and impair mitochondrial function in skeletal muscle[3, 4]. It was therefore to be expected that mitochondrial dysfunction could be a major mechanism underlying exercise intolerance in hypoxia. Given that mitochondrial dysfunction was an important limiting factor for exercise capacity under hypoxic conditions, strategies to prevent mitochondrial impairment and promote mitochondrial repair are predicted to improve exercise performance in hypoxia. Mitochondrial biogenesis is a process by which new and functional mitochondria are formed in the cells, thus mitochondrial dysfunction could be prevented by inducing mitochondrial biogenesis[5–7]. Despite the potential significance of mitochondrial function improvement by maintaining mitochondrial biogenesis in hypoxia-induced exercise intolerance, few strategies to protect mitochondrial biogenesis have been studied to increase exercise tolerance under hypoxic conditions.

Though the genes involved in the regulation of mitochondrial biogenesis are not fully clarified yet, the peroxisome proliferator-activated receptor gamma coactivator-1alpha (PGC-1α) has been shown to be a master regulator of mitochondrial biogenesis[8, 9]. PGC-1α acts as a co-transcriptional regulation factor that induces mitochondrial biogenesis by activating nuclear respiratory factors NRF1 and NRF2, which activate mitochondrial transcription factor A (TFAm), essential for replication, maintenance, and transcription of mitochondrial DNA[10–13]. Therefore, approaches to increase PGC-1α activity directly or indirectly have shown great promise in enhancing mitochondrial biogenesis[14, 15]. Exercise training is generally thought to be the best way to increase muscle mitochondrial biogenesis and function. Given the difficulty in maintaining a regular exercise, other strategies involving nutrition and drugs have received increasing attention[15–17]. Previous studies have demonstrated that quercetin, a natural flavonoid, could increase mitochondrial biogenesis through up-regulation of PGC-1α and sirtuin 1 (SIRT1) and improve physical performance under normoxic conditions[18, 19]. Myricetin (3,5,7,3′,4′,5′-hexahydroxyflavone) is another natural flavonoid with similar chemical structure to quercetin, commonly found in natural foods such as teas, berries, vegetables and medicinal herbs. A recent study revealed that, both quercetin and myricetin could attenuate oxygen-glucose deprivation-induced cell swelling in glial cells, but lower concentrations were required for myricetin because of its effect on mitochondria being more potent[20]. Therefore, we hypothesized that myricetin would possess protective effects against acute hypoxia-induced mitochondrial impairment and exercise intolerance through maintaining mitochondrial biogenesis in muscle.

In the present study, the effects of myricetin on acute hypoxia-induced exercise intolerance and mitochondrial impairments in rats were investigated. Moreover, the actual mechanisms of myricetin were also elucidated via *in vivo* and *in vitro* experiments, including the changes in mitochondrial biogenesis in skeletal muscle and the role of AMP-activated protein kinase (AMPK) and SIRT1 which have been shown to activate PGC-1α[21] in the regulation of mitochondrial biogenesis.

## Materials and Methods

### Ethics Statement

All animal procedures used in this study were approved by The Laboratory Animal Welfare and Ethics Committee of the Third Military Medical University (Permit Number: SYXK-PLA-20120031). All surgery was performed under appropriate anesthesia, and all efforts were made to minimize suffering.

### Animals and treatments

Male Sprague—Dawley rats (180–220 g), obtained from the Experimental Animal Center of the Third Military University, were housed in a controlled environment (23 ± 2°C) with a 12h light-dark cycle (lights on from 06:00 to 18:00). Rats were bred and maintained in accordance with our institutional guidelines and animal welfare for the use of laboratory animals. Food and water were provided ad libitum. Body weight, food and water intake were measured once every two days. Daily animal health checks were performed throughout the study and the animals were reported to be alert, active, and in good health before experimental procedures. Rats were supplied with the following reagents for 7 days after being divided into eight groups (n = 12 each): a normoxia group with placebo (NOR), a control group with placebo (CON), three normoxia-myricetin groups (50, 75, and 100mg/kg per day; termed N-50, N-75, and N-100, respectively) and three hypoxia-exposure-myricetin groups (50, 75, and 100mg/kg per day; termed H-50, H-75, and H-100, respectively). Each dosage was diluted in 1 ml distilled water and then administered via the intra-gastric route, with distilled water used as a placebo. During 7 days of drug treatment, all groups were familiarized with treadmill running for 10 minutes/day.

### Exposure to hypobaric hypoxia

With the exception of normoxia-related groups (including NOR, N-50, N-75, and N-100), other groups were placed in a specially designed hypobaric chamber to expose to a simulated altitude of 5000 m (10.9% oxygen) for 24 h where altitude could be maintained by reducing the ambient barometric pressure. Fresh air was continuously flushed to prevent accumulation of carbon dioxide at a rate of 6 L/min within the chamber. The temperature and humidity in the chamber were maintained at 25±2°C and 58±3% respectively. The ascending period to reach the simulated altitude of 5000 m and the descending period back to sea level conditions lasted for 30 min each. After the 24 h acute hypobaric hypoxic exposure, their endurance capacity was measured (all 8 groups), as described below.

### Run-to-fatigue procedure and muscle tissue preparation

Physical performance was assessed with the run-to-fatigue procedure on a motorized treadmill. Rats were run on a motorized treadmill at a speed of 9 m/min until they reached fatigue, which was defined as the inability to run despite continuous electrode-slice prodding for 20 s. The run-to-fatigue time was recorded. Rats with signs of injury were immediately removed from the treadmill and given appropriate medical treatment. There were no unintended deaths of rats during the whole experimental procedures.

The rats were immediately sacrificed by cervical dislocation after the run-to-fatigue procedure. At the time of sacrifice, the animals were lightly anaesthetized with an intraperitoneal injection of tiletamine and zolazepam (50 mg/kg body weight) as well as xylazine (15 mg/kg body weight). The whole gastrocnemius muscles were carefully isolated from the calf region of the hind limb of the rats, and the red portion (derived from the deep part of the lateral head) was then dissected and used as samples. The red gastrocnemius muscle was chosen because it is primarily composed of oxidative ((types I and IIA) myofibres which contain a high concentration of mitochondria and use oxidative metabolism as a chief energy source. One part of the samples was immediately processed for transmission electron microscopy (TEM) observation or isolation of mitochondria, another part of the samples was fixed in 4% paraformaldehyde at 4°C overnight, embedded in paraffin, and sectioned at a thickness of 5 μm for immunohistochemistry, and the other parts were frozen and stored at -80°C until further analysis.

### TEM observation

Mitochondrial morphology in the gastrocnemius muscle was assessed by TEM. Briefly, muscles were first cut into pieces approximately 1 mm^3^, placed in ice-cold fixative (2.5% glutaraldehyde in 0.1 M sodium cacodylate buffer, pH 7.4) and subsequently in 1% osmium tetroxide, dehydrated in ethanol, block-stained with uranyl acetate, and embedded in Epon in a longitudinal or transverse orientation. Ultrathin sections (60 nm) were doubly stained with 2% uranyl acetate and lead citrate and examined under a TECNAI 10 (Philips, Netherlands) transmission electron microscope at an accelerating voltage of 80 kV. For morphometric studies, randomly selected areas of tissue derived from three animals per group were photographed at 17,500× magnification.

### Isolation of skeletal muscle mitochondria

The isolation procedure was performed on fresh tissues. Skeletal muscle tissue was homogenized using PT 1300D (Brinkmann, Westbury, NY, USA) homogenizer with 12 mm rotor/stator head. The homogenate was centrifuged at 1,000 g for 10 min, and the supernatant was centrifuged again at 12,000 g for 10 min. Mitochondrial pellet was washed twice, centrifuged at 12,000 g, and resuspended in isolation buffer without EGTA. Protein concentration was determined using the BCA Protein Assay kit (Beyotime, Shanghai, China).

### Respiratory enzyme activities

Activities of mitochondrial electron transport chain complex I, II, IV, and V were assessed in mitochondria isolated from gastrocnemius muscle samples of all groups of rats, using an enzyme-linked immuno-sorbent assay kit (ELISA; MitoSciences, Eugene, OR, USA) according to the manufacturer's instructions.

### Quantification of mtDNA

Quantification of mtDNA copy number was performed by real-time PCR. mtDNA was isolated from gastrocnemius muscle tissues and L6 cells using a Mito DNA Extraction Kit (Genmed Scientifics Inc., USA). DNA was quantified spectrophotometrically (260 nm) and subjected to quantitative real-time PCR (100 ng/reaction; SYBR Green I fluorescent RT-PCR protocol, PE Biosystems) in a single color real-time PCR detection system (BIO-RAD, USA). Relative amounts of mtDNA were determined by comparing their amplification products to those of β-actin (forward: 5'-CCACCATGTACCCAGGCATT-3'; reverse: 5'-CGGACTCATCGTACTCCTGC-3') and cytochrome b (forward: 5'- AACGCCAACCCTAGACAACC -3'; reverse: 5'- GAGATGTTAGATGGGGCGGG -3').

### Western blotting

Gastrocnemius muscle from *in vivo* studies were homogenized in a glass homogenizer with SDS lysis buffer (Beyotime Institute of Biotechnology, China) and protease inhibitors (Roche). Muscle homogenates were then centrifuged at a speed of 12000rpm, and the resulting supernatant was transferred to a new tube. For *in vitro* studies, cells were washed in PBS and cellular proteins were extracted in RIPA buffer (Biomed, China) for 30 min at 4°C. Lysates were cleared by centrifugation, and the resulting supernatant was transferred to a new tube. The protein concentration of the supernatant (*in vivo* and *in vitro*) was quantified with the BCA Protein Assay Reagent Kit (Beyotime Institute of Biotechnology, China). Samples were separated using SDS-polyacrylamide gel electrophoresis (PAGE) and transferred to nitrocellulose membranes. The membranes were blocked with 5% non-fat dry milk in Tris-buffered saline Tween-20 (TBST) for 2 h, washed, and probed with the respective polyclonal antibodies. The primary antibodies were anti-mitochondrial transcription factor A (TFAm; 1:2000, ab131607, Abcam, USA), anti-peroxisome proliferator-activated receptor-γ coactivator 1α (PGC1-α; 1:500, ab106814, Abcam, USA), anti—AMP-activated protein kinase (AMPK; 1:2000, ab32047, Santa Cruz, USA), anti-phospo AMPK (p-AMPK; 1:1000, ab72845, Abcam, USA), anti-sirtuin 1 (SIRT1; 1:1000, #9475, Cell Signaling, USA), and anti-nuclear respiratory factor 1 (NRF-1; 1:250, MAB5306, R&D System, Inc, USA). After incubation with the antibody, the membrane was washed with TBST (0.1%) and incubated with 1:2000 dilution peroxidase-conjugated secondary antibodies (Obtained from Boster, China) for 1 h. Proteins were immune-detected using the Fusion fx5 Molecular Imager (Vilber Lourmat, France) and Fusion-capt software. The intensities of the bands were quantified with MCID Analysis 7.0 (http://www.mcid.co.uk/software/mcid_analysis/ InterFocus Imaging, Linton, Cambridge, UK) software.

### Immunohistochemistry

The primary antibodies used were identical to those used for western blot analysis. The 5-μm sections were deparaffinized, hydrated, pretreated with 3% H_2_O_2_ in methanol, and then treated with citrate (10 mmol/L) for antigen retrieval. Sections were blocked in 2% fetal bovine serum before incubation with primary antibodies overnight at 4°C. Sections were then incubated with biotinylated IgG antibodies at room temperature for 1 h. Next, slides were treated with ABC reagents (Boster, Wuhan, China), and signals from antibody binding visualized with diaminobenzidine (Boster) substrate. Counterstaining was performed with hematoxylin. Slides were fully rinsed with 0.1 M PBS or distilled water between individual steps. To obtain the percentage of cells expressing a given marker protein, photomicrographic images of each section were captured with an Olympus microscope and digital camera. The number of specific antigen-positive cells was counted in five random fields. The mean and standard error of the number of positive cells were calculated for each group and were used for the statistical analysis.

### Cell culture and treatments

The rat L6 myogenic cell line (obtained from ATCC-CRL-1458; USA) was grown in Dulbecco’s Modified Eagle Medium (DMEM; Gibco-Invitrogen, Carlsbad, CA) with 10% fetal bovine serum (FBS; Hyclone, USA) and supplemented with penicillin (120 units/ml), streptomycin (75 μg/ml), gentamycin (160 μg/ml), and amphotericin B (3 μg/ml) in a 5% CO_2_ environment. For differentiation, the L6 cells were transferred to DMEM with 2% FBS for seven days. The extent of differentiation was established by assaying the gene expression of myogenin and myosin heavy chain (MHC), markers of L6 cell differentiation, by real-time PCR as previously described[22]. The results demonstrated that the L6 cells were fully differentiated. Differentiated L6 cells, given fresh media and reagents for 24 hours, were divided into five groups: a normoxia group (NORc) with vehicle, a control group (CONc) with vehicle, a myricetin group (10 μmol/L, this concentration was used based on previous results by Huang et al.[23]; MC), a SIRT1-inhibited myricetin group (SI-MC) with both myricetin(50 μmol/L) and the SIRT1 inhibitor nicotinamide (5 mmol/L; Abcam, USA), an AMPK-inhibited myricetin group (AI-MC) with both myricetin (50 μmol/L) and the AMPK inhibitor dorsomorphin dihydrochloride (20 μmol/L; Santa Cruz, USA). After the drug pre-treatment, L6 cells (without NORc) were exposed to hypoxia as previously reported[24]. Briefly, cultures were transferred into an automatically-controlled Multi Gas Incubator (YCP-50S, Hua-xi, Changsha, China) in which oxygen level (3% O_2_, 5% CO_2_, and 92% N_2_) and temperature (37°C) were controlled. Cells then remained in the hypoxic incubator for 16 h incubation periods. Untreated control cells (NORc) were not exposed to hypoxia.

### Immunofluorescence staining

L6 cells were stained using Mitotracker Red CMXRos (CST. Co. USA) immediately after exposure to hypoxia. Cells were then fixed in 4% paraformaldehyde for 30 min at 22°C, followed by permeabilization with 0.1% Triton for 20 min at 22°C. After rinsing three times with PBS, cells were incubated in a blocking solution (5% BSA; Beyotime, China) for 1 hour at 37°C and then incubated with the appropriate primary antibodies overnight at 4°C. After washing with PBS, cells were subsequently incubated for 1 hour with anti-rabbit, anti-mouse or anti-goat IgG antibodies conjugated to a fluorochrome, and then stained with DAPI (Beyotime, China) to visualize nuclei. Confocal laser scanning microscopy was carried out with the LSM 780 NLO laser scanning microscope (Carl Zeiss, Germany). The images presented were captured under constant exposure time, gain, and offset. Quantification of relative amounts of fluorescently labeled proteins in intracellular compartments was analyzed by the ImarisXT software (Bitplane).

### Measurement of mitochondrial membrane potential

Mitochondrial membrane potential (ΔΨm) was measured by 5,5′,6,6′-tetrachloro-1,1′,3,3′-tetraethylbenzimidazole-carbocyanide iodine (JC-1; Beyotime, China) staining as previously described[25]. Briefly, after drug treatment and the hypoxic exposure, L6 cells were incubated with an equal volume of 5 mg/L JC-1 staining solution for 20 min at 37°C. Images were then viewed and scanned by a confocal laser microscope (TCS-SP5, Leica, Germany) at 514 nm excitation and 529 nm emission for green, and at 585 nm excitation and 590 nm emission for red. Red emission of the dye represented a potential-dependent aggregation in the mitochondria, reflecting ΔΨm. Green fluorescence, appearing in the cytosol after mitochondrial membrane depolarization, represented the monomeric form of JC-1. The fluorescence intensity was detected with a monochromator microplate reader (Safire II, Tecan, Switzerland). The ΔΨm of L6 cells in each treatment group were calculated as the ratio of red/green fluorescence. In addition, cell treated with 10 μM carbonyl cyanide m-chlorophenylhydrazone (CCCP) was used as negative control. CCCP is a protonophore that can dissipate ΔΨm.

### Statistical analyses

Statistical significance was determined using one-way ANOVA and multiple comparison between the groups was performed using S-N-K method. SPSS 11.0 (SPSS Inc., Chicago, IL, USA) was used to analyze the data. Data were expressed as means ± SEM. Statistical significance was set at *P*<0.05.

## Results

### Myricetin improves exercise performance in acute hypoxia

**Fig 1:**
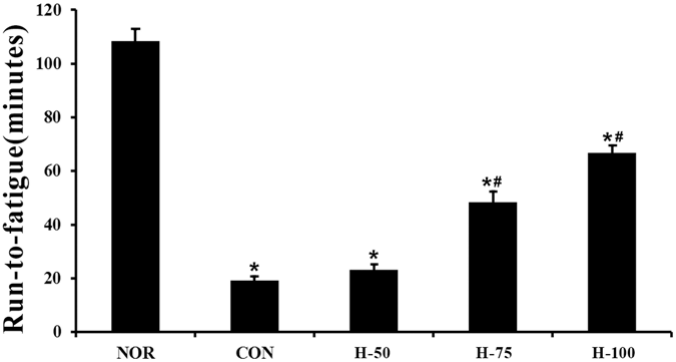
Effect of myricetin on exercise capacity in acute hypoxia. Rats were pretreated with myricetin or placebo for 7 days, then the run-to-fatigue procedure under acute hypoxia exposure was performed, and the run-to-fatigue time of each group was recorded (n = 12/group). Values are means±SEM. *Significantly different from normoxia group (P<0.05). #Significantly different from control group (P<0.05).

First, the effect of myricetin on exercise performance of rats under acute hypoxic conditions was assessed by the run-to-fatigue experiment. The average run-to-fatigue time in the hypoxic group dramatically decreased compared with that in normoxic rats (Fig 1, *P*<0.05), indicating exercise tolerance was severely impaired in acute hypoxia. Meanwhile, the results also showed that rats treated with myricetin at the dose of 75 or 100 mg/kg but not 50 mg/kg for 7 days had a significantly longer run-to-fatigue time than the vehicle-treated control rats (Fig 1, *P*<0.05), suggesting that myricetin supplementation could effectively improve acute hypoxia-induced exercise intolerance in rats.

### Myricetin attenuates acute hypoxia-induced mitochondrial impairment in skeletal muscle cells *in vivo* and *in vitro*

**Fig 2:**
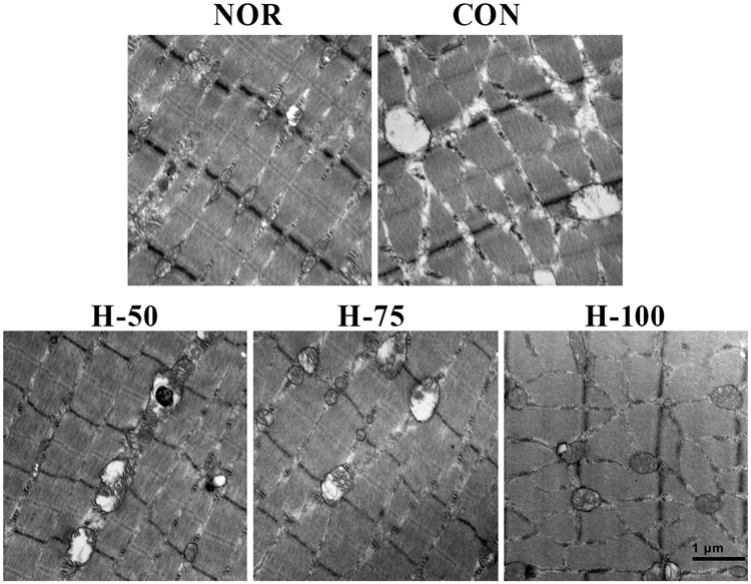
Representative TEM micrographs of mitochondria in skeletal muscle cells (17,500× magnification). In NOR group, the structure of mitochondria and its cristae is intact; in CON, H-50 and H-75 groups, swollen mitochondria with disorganized and fragmented cristae were observed; in H-100 group, mitochondrial swelling and cristae disarray were greatly attenuated.

Maintenance of morphology is essential for normal mitochondrial function. We observed the effects of myricetin on mitochondrial morphology in gastrocnemius muscles of run-to-fatigued rats under acute hypoxic conditions at the ultrastructural level through transmission electron microscopy. As shown in Fig 2, acute hypoxia exposure resulted in swollen mitochondria with disorganized and fragmented cristae, while myricetin supplementation attenuated mitochondrial swelling and cristae disarray.

**Fig 3:**
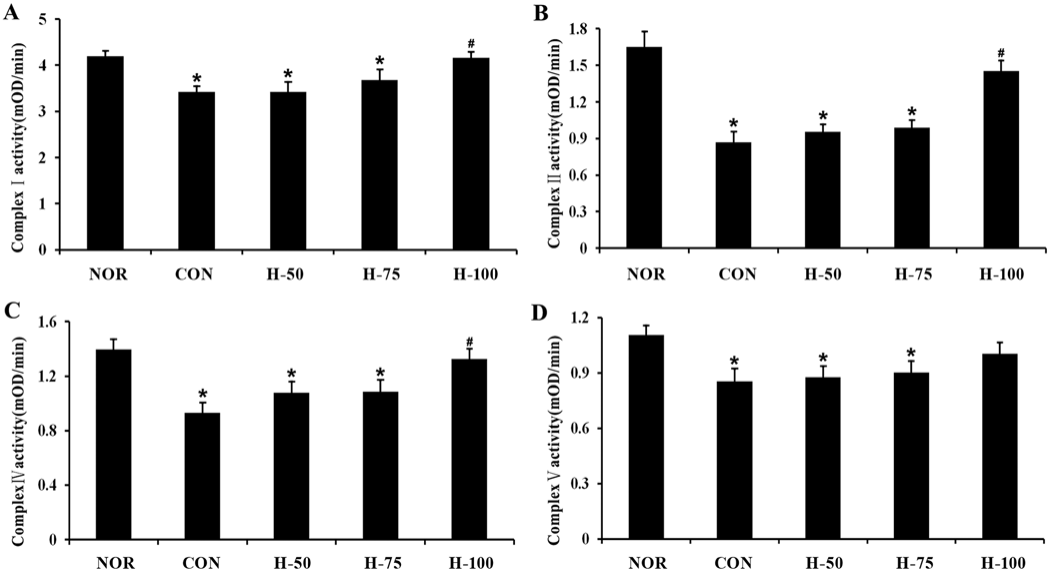
Effect of myricetin on the activities of mitochondrial respiratory chain complexes under acute hypoxic exposure. (A) Complex I, (B) complex II, (C) complex IV, and (D) complex V. Data are expressed as mean±SEM. *Significantly different from normoxia group (P<0.05). #Significantly different from control group (P<0.05).

Mitochondrial morphological abnormalities should be accompanied by alterations in mitochondrial function. Mitochondrial function was evaluated by examining the changes in its membrane potentials and the activities of mitochondrial respiratory chain complexes. In vivo study, acute hypoxic exposure caused a significant decrease in the activities of mitochondrial complexes I, II, IV, and V in gastrocnemius muscle of rats, while treatment with myricetin significantly improved the activities of mitochondrial complexes (I, II, IV) compared with the control (Fig 3; *P*<0.05).

**Fig 4:**
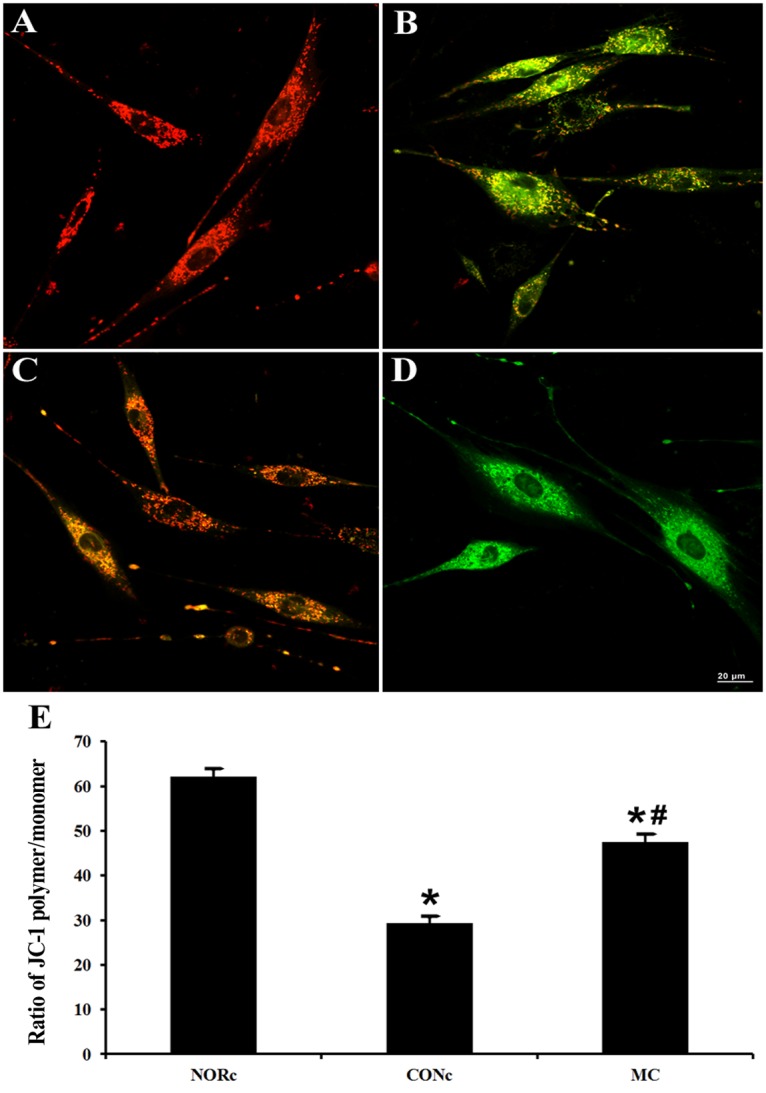
Effect of myricetin on mitochondrial membrane potential (ΔΨm) in the L6 muscle cell line. Red fluorescence represents the mitochondrial aggregate form of JC-1, indicating intact mitochondrial membrane potential. Green fluorescence represents the monomeric form of JC-1, indicating dissipation of ΔΨm. (A) Normoxia cells, (B) hypoxia cells, (C) myricetin-treated hypoxia cells, (D) CCCP treated cells. (E) ratio of red fluorescence to green fluorescence. Data are expressed as mean±SEM. *Significantly different from normoxia group (P<0.05). #Significantly different from control group (P<0.05).

Furthermore, *in vitro* study, the effects of myricetin treatment on mitochondrial membrane potential (ΔΨm) in rat L6 cells under acute hypoxic conditions were measured by using the fluorescent probe JC-1. Results showed that the ratio of JC-1 red to green fluorescence in L6 cells significantly decreased after exposure to hypoxia for 16 h (Fig 4, *P*<0.05), indicating acute hypoxia resulted in dissipation of ΔΨm. However, Myricetin treatment could significantly prevent hypoxia-induced dissipation of ΔΨm in L6 cells (Fig 4, *P*<0.05).

Taken together, these results revealed that acute hypoxic exposure significantly impaired skeletal muscle mitochondria as evidenced by morphological abnormalities and mitochondrial dysfunction including decreases in respiratory chain complexes activities and dissipation of ΔΨm, and these impairments could be effectively attenuated by myricetin treatment.

### Myricetin maintains mitochondrial biogenesis in skeletal muscle cells under hypoxia

**Fig 5:**
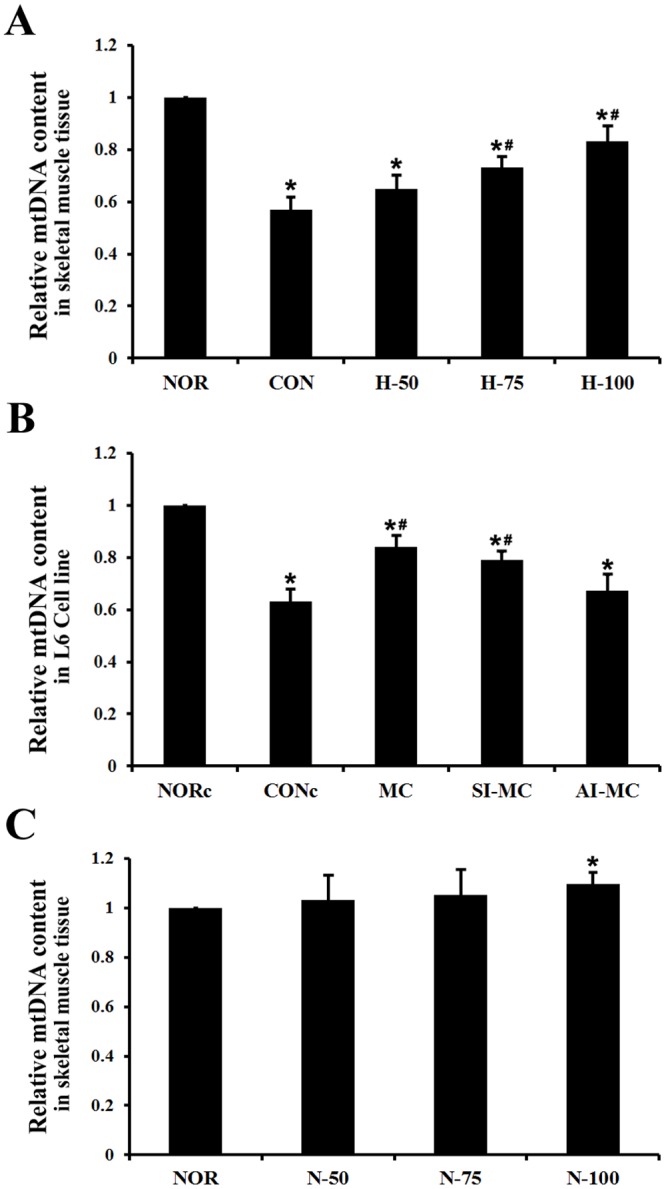
Effect of myricetin on mtDNA content. (A) The relative mtDNA amounts were measured in skeletal muscle tissue of rats under acute hypoxia exposure. (B) The relative mtDNA amounts were measured in the L6 muscle cell line under acute hypoxia. (C) The relative mtDNA amounts in skeletal muscle tissue of rats under sea level conditions. Data are expressed as mean±SEM. *Significantly different from normoxia group (P<0.05). #Significantly different from control group (P<0.05).

Mitochondrial biogenesis as a process by which new and functional mitochondria are generated in the cells, plays a pivotal role in the replacement of impaired mitochondria[26]. Therefore, we further investigated whether acute hypoxia could result in mitochondrial biogenesis impairment and observed the effect of myricetin supplementation on mitochondrial biogenesis. We first examined the mtDNA content as a biomarker of mitochondrial biogenesis in gastrocnemius muscle of rats following exposure to acute hypoxia. As shown in Fig 5A, the amount of mtDNA severely decreased after acute hypoxic exposure (*P*<0.05). However, pretreatment with myricetin effectively prevented the decline of the mtDNA content compared with the control, myricetin supplementation at the dose of 50, 75 and 100 mg/kg increased mtDNA amount by 13.8%, 28% and 45.8%, respectively. Studies in L6 cells showed similar results (Fig 5B, *P*<0.05).

**Fig 6:**
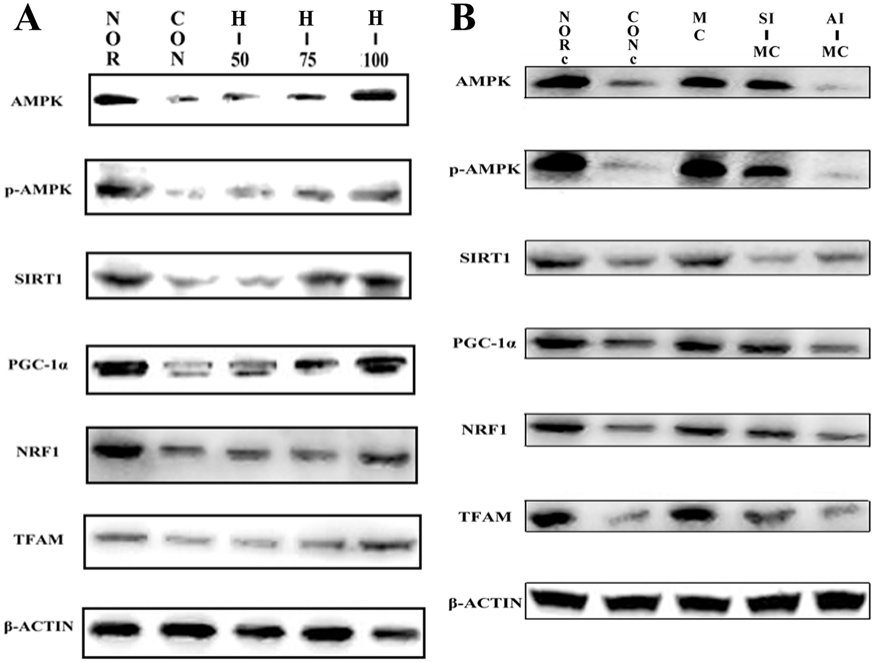
Effect of myricetin on mitochondrial biogenesis regulators under acute hypoxia detecting by western blotting. (A) Representative images of western blots for genes related to mitochondrial biogenesis in skeletal muscle tissue of rats under acute hypoxia exposure; (B) Representative images of western blots for genes related to mitochondrial biogenesis in L6 muscle cell line.

**Fig 7:**
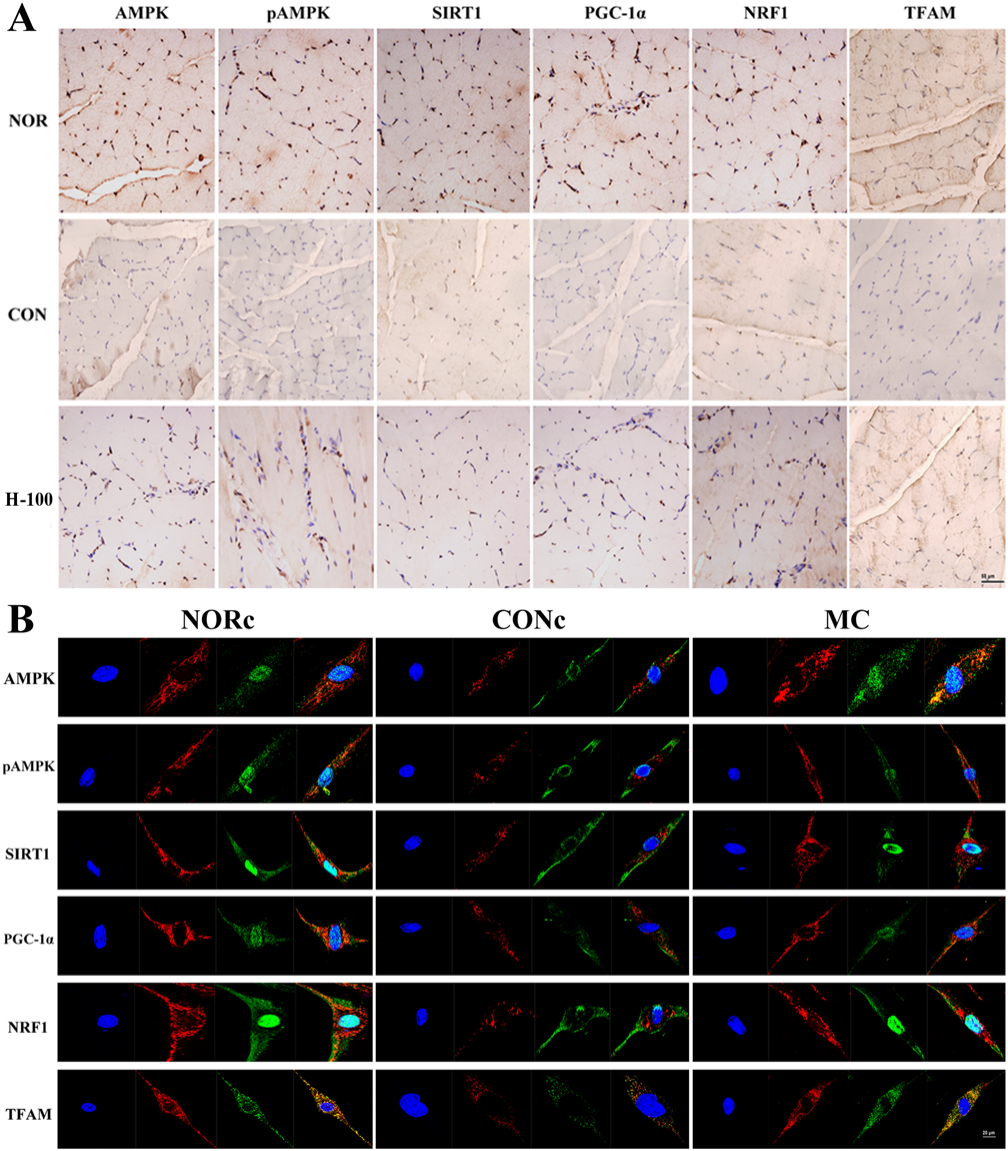
Mitochondrial biogenesis regulators expression under acute hypoxia in vivo and in vitro. (A) Representative images of immunohistochemistry of proteins related to mitochondrial biogenesis in skeletal muscle tissue (400× magnification). (B) Representative confocal microscopic images of immunofluorescence staining of proteins related to mitochondrial biogenesis in L6 muscle cell line. Blue fluorescence represents DAPI staining; Red fluorescence represents the Mitotracker indicating location of mitochondria; Green fluorescence represents the targeted proteins expression; Yellow shows the merged image.

Because PGC-1a has shown to be a master regulator of mitochondrial biogenesis, we next assessed the effect of acute hypoxia exposure and myricetin treatment on the expression of PGC-1α protein in gastrocnemius muscle and L6 cells. Western blot analysis revealed that PGC-1α protein expression was significantly down-regulated both in gastrocnemius muscle and L6 cells, while myricetin supplementation effectively rescued this down-regulation (Fig 6, S1A–S1D Fig, *P*<0.05). Moreover, the Western blot findings were confirmed by immunohistochemical analysis (Fig 7A, S1E Fig, *P*<0.05) and immunofluorescence experiments (Fig 7B, S1F and S1G Fig, *P*<0.05).

NRF-1 and Tfam are located downstream of PGC-1a as co-activating targets involved in mitochondrial biogenesis. Therefore, we further measured the expression of these important genes involved in mitochondrial biogenesis. As shown in Figs 6 and 7 and S1 Fig, we found that the protein expression of NRF-1 and Tfam significantly decreased in gastrocnemius muscle and L6 cells after acute hypoxic exposure (*P*<0.05), while myricetin treatment obviously increased the protein expression of NRF-1 and Tfam compared with that of acute hypoxia exposure alone (*P*<0.05).

These data showed potent effects of myricetin on muscle mitochondrial biogenesis in hypoxia. However, it is unclear whether the induction of mitochondrial biogenesis by myricetin prior to hypoxic exposure resulted in higher mitochondrial content after the hypoxic insult, thus, we investigated the effects of myricetin on mtDNA content and mitochondrial biogenesis parameters of skeletal muscle under normoxic conditions. The results showed that myricetin supplementation at the dose of 50, 75 and 100 mg/kg increased mtDNA amount by 3.2%, 5.2% and 9.7%, respectively (Fig 5C), and western blots assays confirmed myricetin promoted mitochondrial biogenesis parameters (S2 Fig, *P*<0.05). Obviously, the enhancement effects of myricetin on mtDNA content under acute hypoxia exposure were significantly greater than that under normoxia conditions, suggesting that myricetin remains inducing mitochondrial biogenesis under hypoxic conditions.

### AMPK plays a crucial role in the protective effects of myricetin on mitochondrial biogenesis in skeletal muscle cells

It is well-established that AMPK and SIRT1 regulate PGC-1α activity, resulting in increased mitochondrial biogenesis[27, 28]. Therefore, we first investigated the effects of acute hypoxia exposure and myricetin treatment on the expression of AMPK and SIRT1. As shown in Figs 6 and 7 and S1 Fig, we found that acute hypoxia resulted in the decrease of the protein expression of SIRT1, phosphorylated AMPK and total AMPK in gastrocnemius muscle and L6 cells, which were significantly attenuated by myricetin treatment (*P*<0.05).

To further determine the role of AMPK and SIRT1 in the prevention of myricetin against acute hypoxia-induced mitochondrial biogenesis impairment in skeletal muscle, L6 cells were pretreated with specific inhibitors of SIRT1 and AMPK respectively before myricetin treatment. As shown in Figs 5B and 6B and S1F and S1G Fig, The AMPK inhibitor compound C significantly compromised the effects of myricetin on the expression of mitochondrial biogenesis markers and mtDNA content, while the SIRT1 inhibitor showed no apparent influence (*P*<0.05). These findings suggested that activation of AMPK, but not SIRT1, was the initial factor for maintaining mitochondrial biogenesis by myricetin.

## Discussion

Under acute exposure to high altitude hypoxia, exercise performance is decreased and the primary factor contributing to this decrement is reduced atmospheric oxygen pressure[29–32]. However, it should not be ignored that skeletal muscle mitochondria also play an important role in exercise tolerance as mitochondria are the major site of cellular ATP production. In fact, studies in adult rodents have shown that either brief or prolonged exposure to hypobaric hypoxia could induce mitochondrial impairment from oxidative stress and lead to a modest down-regulation in oxidative phosphorylation capacity[3, 33]. Therefore, hypoxia-induced mitochondrial dysfunction also contributes to the reduced exercise tolerance in acute hypoxia, which raises the question of whether mitochondrial dysfunction could be pharmacologically prevented to preserve exercise capacity under hypoxic conditions.

Myricetin was tested in this study for three reasons: first, myricetin as a common dietary flavonoid possesses potent antioxidative activity with very little side effects[34, 35], while oxidative stress is involved in hypoxia-induced mitochondrial dysfunction and antioxidant vitamin E has been proven helpful for preventing mitochondrial dysfunction in skeletal muscle[3, 4]; second, early studies indicated that quercetin, a flavonoid with similar chemical structure to myricetin, is responsible for improving exercise performance by increasing mitochondrial biogenesis under normoxic conditions[18, 19]; third, one study found that in simulated ischemic conditions, myricetin was more potent than quercetin on attenuating cell swelling in glial cells by acting on the mitochondria[20]. However, little attention has been paid to evaluating the biological activities of myricetin on hypoxia-induced mitochondrial impairment and exercise intolerance. The results of this study support the positive role of myricetin in mitochondrial dysfunction. In the exercise performance test under acute hypoxic condition, myricetin pretreatment increased run-to-fatigue time in a dose-dependent manner. Furthermore, the mechanism underlying the protective effect of myricetin was found to impact mitochondrial biogenesis in skeletal muscle.

Preservation of mitochondrial structure and function in skeletal muscle is essential for maintaining exercise performance. Therefore, we determined whether the Protective effects of myricetin on exercise intolerance were associated with an improvement in mitochondrial structure and function. Ultrastructural observation by TEM indicated that myricetin treatment could attenuate skeletal muscle mitochondrial swell and cristae disarray induced by acute hypoxia exposure and exhausted exercise. Mitochondria membrane potential (ΔΨm) is generated by a proton gradient with the electron transfer of mitochondrial respiratory chain, thus inhibition of mitochondrial respiratory chain complexes would result in decrease in ΔΨm, which in turn impair ATP synthesis. Therefore, loss of ΔΨm is a indicator of mitochondrial dysfunction. In the present study, we found that hypoxia inhibited the activities of mitochondrial respiratory chain complexes and caused dissipation of ΔΨm in skeletal muscle cells, while these changes could be ameliorated by myricetin supplementation. The results suggested that myricetin protects against hypoxia-induced exercise intolerance through preserving mitochondrial structure and function.

Inducing mitochondrial biogenesis has been reported to improve mitochondrial dysfunction[7]. This study examined the effects of myricetin treatment on the mtDNA content as a biomarker of mitochondrial biogenesis and the expression of several genes involved in the regulation of mitochondrial biogenesis, including PGC-1α and some of its downstream targets, such as NRF-1 and Tfam. The data indicate that myricetin supplementation could prevent decreases in the mtDNA content and the protein expression of PGC-1α, NRF-1 and Tfam in skeletal muscle induced by acute hypoxia exposure, which reflect impairment of mitochondrial biogenesis. PGC-1α has been reported to play an important role in enhancing mitochondrial biogenesis[8]. NRF-1 and Tfam are downstream conductors of PGC-1α in the regulation of mitochondrial biogenesis[36–38]. The overexpression of PGC-1α in rodent skeletal muscle produces a phenotype remarkably similar to aerobically-trained muscle, illustrated by increases in mitochondrial density, respiratory capacity, ATP synthesis, and improved exercise performance[39, 40]. As a result of increases in mitochondrial biogenesis, increases in muscle mtDNA have benefits on exercise tolerance. Furthermore, we found that myricetin had a relative stronger induction of mitochondrial biogenesis under acute hypoxia exposure as compared to normoxic conditions (Fig 5A and 5C), it suggested that myricetin still induce mitochondrial biogenesis under hypoxic conditions. Thus, we conclude that myricetin could attenuate hypoxia-induced impairment of mitochondrial biogenesis. However, the induction of mitochondrial biogenesis prior to hypoxic exposure might also contribute to the protective effect of myricetin.

AMP-activated protein kinase (AMPK) and SIRT1 have been reported to directly regulate PGC-1a activity through phosphorylation and deacetylation, respectively[27, 41–43]. Therefore, the roles of AMPK and SIRT1 in the regulation of PGC-1α expression by myricetin were also investigated in this study. The results revealed that the protein expressions of AMPK and SIRT1 were significantly decreased in skeletal muscle cells both in vivo and in vitro after acute hypoxic exposure, and that myricetin pretreatment could obviously reverse this down-regulation, indicating AMPK and SIRT1 may be correlated with the preventive effects of myricetin on hypoxia-induced reduction of mtDNA content and PGC-1α, NRF-1 and Tfam expression in skeletal muscle. However, in our in vitro study, the protective effects of myricetin on mitochondrial biogenesis in L6 cells could be abrogated by AMPK-specific inhibitor compound C but not SIRT1-specific inhibitor nicotinamide, suggesting that AMPK, but not SIRT1, plays a key role in the prevention of myricetin against acute hypoxia-induced mitochondrial biogenesis impairment. However, a previous study showed that SIRT1 is a downstream target of AMPK[44]. In addition, the possible mechanisms by which myricetin acts to maintain mitochondrial biogenesis and function were summarized as a schematic in S3 Fig.

In conclusion, our results are the first to demonstrate that the supplementation of myricetin before acute hypoxic exposure may be beneficial to preserve exercise capacity by counteracting mitochondrial dysfunction t through inducing mitochondrial biogenesis both prior to and during hypoxic exposure. Furthermore, AMPK plays a crucial role in this process. Therefore, myricetin is an effective natural agent that prevents acute hypoxia-induced exercise intolerance and potentially other altitude-related illnesses.

## Supporting Information

S1 Fig Quantitative analysis of mitochondrial biogenesis regulators expression under acute hypoxia in vivo and in vitro. (A) (C) Relative protein expression of AMPK, pAMPK and SIRT1 in skeletal muscle tissue and in L6 myogenic cell line detecting by western blotting. (B) (D) Relative protein expression of PGC-1α, NRF1 and TFAM in skeletal muscle tissue and in L6 myogenic cell line detecting by western blotting. (E) Number of Immunohistochemistry positive cells of mitochondrial biogenesis regulators in skeletal muscle tissue. (F) (G) Quantification of relative amounts of fluorescently labeled proteins positive cells of mitochondrial biogenesis regulators in L6 myogenic cell line. Data are expressed as mean±SEM. *Significantly different from normoxia group (P<0.05). #Significantly different from control group (P<0.05).
(TIF)

S2 Fig Effect of myricetin on mitochondrial biogenesis regulators under normoxia conditions detecting by western blotting. (A) Representative images of western blots for genes related to mitochondrial biogenesis and (B) their quantitative analysis in skeletal muscle tissue of rats normoxia conditions. Data are expressed as mean±SEM. *Significantly different from normoxia group (P<0.05). #Significantly different from control group (P<0.05).
(TIF)

S3 Fig Schematic representation of the possible mechanism of myricetin acting to maintain mitochondrial biogenesis and function. (TIF)
