# Correction: Protective Effects of Myricetin on Acute Hypoxia-Induced Exercise Intolerance and Mitochondrial Impairments in Rats

**DOI:** 10.1371/journal.pone.0133336

**Published:** 2015-07-15

**Authors:** Dan Zou, Peng Liu, Ka Chen, Qi Xie, Xinyu Liang, Qian Bai, Qicheng Zhou, Kai Liu, Ting Zhang, Jundong Zhu, Mantian Mi

There are multiple errors in the fifth sentence of the “Cell culture and treatments” subsection of the Materials and Methods. The correct sentence is: Differentiated L6 cells, given fresh media and reagents for 24 hours, were divided into five groups: a normoxia group (NORc) with vehicle, a control group (CONc) with vehicle, a myricetin group (10 μmol/L, this concentration was used based on previous results by Huang et al.[23]; MC), a SIRT1-inhibited myricetin group (SI-MC) with both myricetin (10 μmol/L) and the SIRT1 inhibitor nicotinamide (5 mmol/L; Abcam, USA), an AMPK-inhibited myricetin group (AI-MC) with both myricetin (10 μmol/L) and the AMPK inhibitor dorsomorphin dihydrochloride (20 μmol/L; Santa Cruz, USA).
