# Supplementary figures and images for: Protective Effects of Myricetin on Acute Hypoxia-Induced Exercise Intolerance and Mitochondrial Impairments in Rats
Source: PLoS One. 2015 Apr 28;10(4):e0124727. doi: 10.1371/journal.pone.0124727 (PMC4412664; doi:10.1371/journal.pone.0124727)

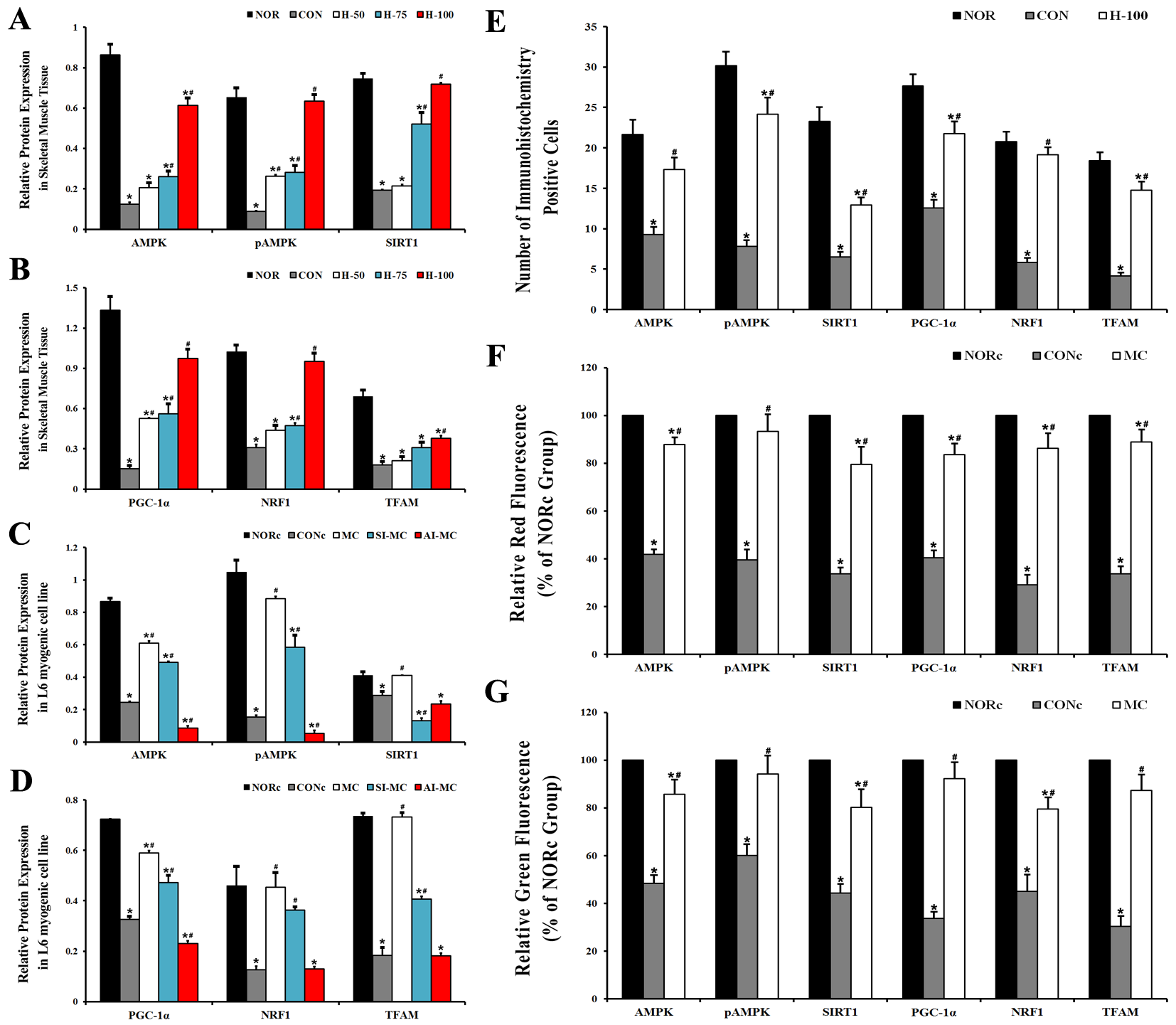

Supplement: S1 Fig — (A) (C) Relative protein expression of AMPK, pAMPK and SIRT1 in skeletal muscle tissue and in L6 myogenic cell line detecting by western blotting. (B) (D) Relative protein expression of PGC-1α, NRF1 and TFAM in skeletal muscle tissue and in L6 myogenic cell line detecting by western blotting. (E) Number of Immunohistochemistry positive cells of mitochondrial biogenesis regulators in skeletal muscle tissue. (F) (G) Quantification of relative amounts of fluorescently labeled proteins positive cells of mitochondrial biogenesis regulators in L6 myogenic cell line. Data are expressed as mean±SEM. *Significantly different from normoxia group (P<0.05). #Significantly different from control group (P<0.05). (TIF) [file pone.0124727.s001.tif]

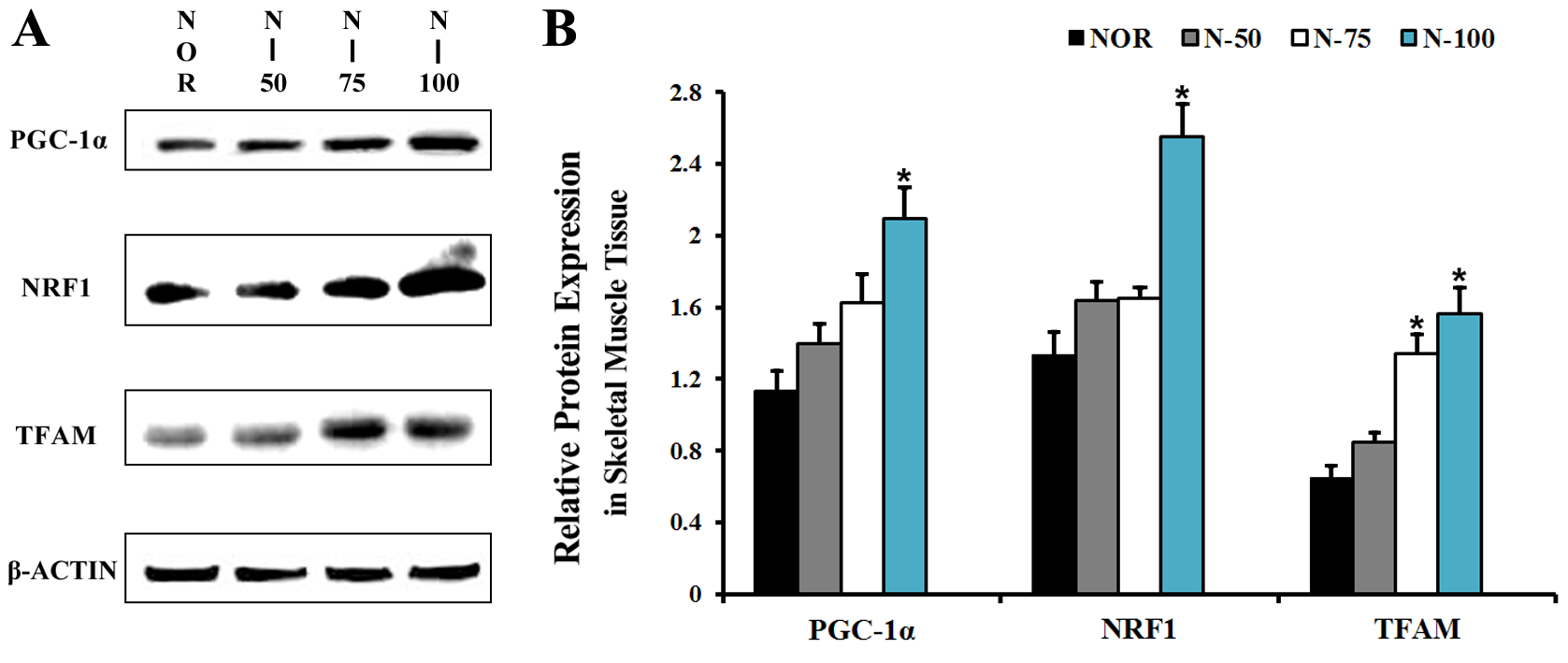

Supplement: S2 Fig — (A) Representative images of western blots for genes related to mitochondrial biogenesis and (B) their quantitative analysis in skeletal muscle tissue of rats normoxia conditions. Data are expressed as mean±SEM. *Significantly different from normoxia group (P<0.05). #Significantly different from control group (P<0.05). (TIF) [file pone.0124727.s002.tif]

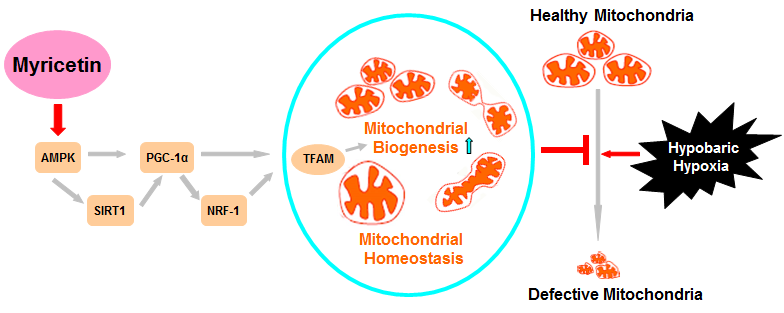

Supplement: S3 Fig — (TIF) [file pone.0124727.s003.tif]
